# Vibration mechanosignals superimposed to resistive exercise result in baseline skeletal muscle transcriptome profiles following chronic disuse in bed rest

**DOI:** 10.1038/srep17027

**Published:** 2015-11-24

**Authors:** Michele Salanova, Guido Gambara, Manuela Moriggi, Michele Vasso, Ute Ungethuem, Daniel L. Belavý, Dieter Felsenberg, Paolo Cerretelli, Cecilia Gelfi, Dieter Blottner

**Affiliations:** 1 Center of Space Medicine Berlin, Neuromuscular System, Institute of Anatomy, Charité Universitätsmedizin Berlin, Germany; 2 Department of Biomedical Sciences for Health, University of Milan, Milan, Italy; 3 Italian Federation of Sport Medicine, CONI, Rome, Italy; 4 Institute of Bioimaging and Molecular Physiology, National Research Council (CNR), Segrate-Cefalù, Italy; 5 Laboratory of Functional Genomics, Charité Universitätsmedizin Berlin, Germany; 6 Center for Muscle and Bone Research (ZMK), Charité Universitätsmedizin Berlin, Germany; 7 IRCCS Policlinico San Donato, San Donato Milanese, Milano, Italy

## Abstract

Disuse-induced muscle atrophy is a major concern in aging, in neuromuscular diseases, post-traumatic injury and in microgravity life sciences affecting health and fitness also of crew members in spaceflight. By using a laboratory analogue to body unloading we perform for the first time global gene expression profiling joined to specific proteomic analysis to map molecular adaptations in disused (60 days of bed rest) human *soleus* muscle (CTR) and in response to a resistive exercise (RE) countermeasure protocol without and with superimposed vibration mechanosignals (RVE). Adopting Affymetrix GeneChip technology we identified 235 differently transcribed genes in the CTR group (end- vs. pre-bed rest). RE comprised 206 differentially expressed genes, whereas only 51 changed gene transcripts were found in RVE. Most gene transcription and proteomic changes were linked to various key metabolic pathways (glycolysis, oxidative phosphorylation, tricarboxylic acid (TCA) cycle, lipid metabolism) and to functional contractile structures. Gene expression profiling in bed rest identified a novel set of genes explicitly responsive to vibration mechanosignals in human *soleus*. This new finding highlights the efficacy of RVE protocol in reducing key signs of disuse maladaptation and atrophy, and to maintain a close-to-normal skeletal muscle quality outcome following chronic disuse in bed rest.

Primary degenerative neuromuscular pathology together with prolonged muscle disuse due to trauma or disease, as well as prolonged exposure to microgravity are the main causes of skeletal muscle atrophy. On the other hand, several diseases such as cardiac failure, chronic obstructive pulmonary disorders, cancer and AIDS are also known to induce a rapid loss of muscle mass and strength^1^,^2^. During spaceflights, the capability of astronauts to manage daily tasks (e.g., facility management control and experimental work) as well as efforts for performing the tasks required by intra and extra vehicular activities may become very demanding.

The decrement of mass and strength in muscle atrophy is associated with an increased protease activity and often accompanied by a reduced rate of protein synthesis^3^. A decrease in skeletal muscle fiber cross sectional area (CSA), a myofiber phenotype shift from slow to fast (type I → Type II), the rearrangement of muscle-specific gene expression, the microstructural and molecular reorganization at the neuromuscular junctions due to changes of their electrolyte micro-environment, are among the most frequent features of such maladaptation processes in extended disuse^4^,^5^.

Bed rest (BR) is a well-accepted on ground-based analogue of unloading-induced musculoskeletal atrophy in healthy humans, for investigating the potential efficacy of exercise countermeasures in a strictly controlled experimental setting. BR particularly affects antigravity muscles of the lower extremities (*soleus*) or back (*multifidus*) used for posture control and body stabilization with a consequent decrease in skeletal muscle activity, mass and energy consumption (for review^1^). To date, numerous bed rest studies of various durations (short-, medium, long-term, i.e. 7−, 21− and 60–90 days of BR) have been performed without or with specific countermeasures such as exercise protocols, as well as nutritional and/or pharmacological interventions^6^. The studies mainly aimed at evaluating the structural, ultrastructural and biochemical changes induced by chronic disuse, and have evidenced some of the specific signalling pathways in muscle wasting during acute and chronic disuse^4^,^7^,^8^,^9^,^10^,^11^.

As of today few studies investigated chronic disuse-atrophy at global gene expression level. For example, Yi-Wen Chen *et al.* analyzed gene expression in the gastrocnemius of patients with ankle fracture 4–11 days after cast immobilization, by microarray gene profiling thus identifying a significant change in the expression level of genes linked to energy metabolism and mitochondrial functions^12^. They observed a down-regulation in the expression of genes involved in proteolysis and in the IGF-1 pathway. The comprehensive gene expression pattern in limb muscle was further analyzed in normal healthy subjects in a medium-term (20 days) bed rest study: an up-regulation of two ubiquitin ligases, Cbl-b and Siah-1A were found^13^. Moreover, Chopard and co-workers^14^ reported the efficacy of exercise-based countermeasures on the differential expression of genes induced by long-term (60 days) bed rest in female limb skeletal muscle by means of microarray analysis^14^. A more recent human study showed that 48 h of unloading was sufficient to induce acute changes in the global expression of genes involved in protein ubiquitination and oxidative stress pathways^15^.

Recently, we scrutinized the efficacy of short-duration and frequency-controlled neuroreflexive stimulation with a high resistance mechano-vibration stimulation protocol (RVE) as a countermeasure in 60 days bed rest (1^st^ BBR-1 study)^11^. In a follow-up bed rest study (2^nd^ BBR-2 study) we next compared RVE to a standard high loading and maximally output resistive exercise (RE) protocol, and found a superior preventive outcome showing reduced morphological and biochemical signs of disuse muscle atrophy^7^,^8^,^16^. Previous proteomic analysis of biopsy samples from the 2^nd^ Berlin BedRest study (BBR-2) showed that both RVE and RE resulted in a differential regulation of various skeletal muscle proteins in the human *soleus* and *vastus lateralis* following chronic bed rest disuse^8^.

However, the more complex molecular adaptive changes observed in disused normal human muscle are still poorly documented at the global gene expression level. Even more so, the muscle-specific molecular targets of vibration mechanical stimulation of human postural muscles are largely unknown. Therefore, in the present study we compared the outcome of RE and RVE exercise countermeasures on the human skeletal muscle transcriptome and, furthermore, validated these data by proteome analysis of biopsy material from the same trained and untrained SOL samples from the BBR2–2 study^16^. We here provide a comprehensive map of the global changes of the human SOL gene expression in bed rest without and with exercise countermeasures.

Functional gene clustering analysis resulted in identification of some of the key structural and metabolic pathways specifically affected by either of the two exercise countermeasure protocols (RE vs. RVE) in bed rest. The detailed novel findings from global gene expression profiling validated in part by proteome analysis allow us now to propose RVE as a more physiological countermeasure protocol than conventional resistive exercise protocols applied in the same study to prevent altered disuse-induced gene expression patterns with potentially adverse effects on structural and functional muscle adaptation to disuse and exercise. In fact, RVE protocol is able to maintain a close to pre-BR condition skeletal muscle quality and may serve as a feasible and effective adjunct to future countermeasure prescriptions to prevent impaired performance by normal subjects during longer periods of disuse in spaceflight^17^, sarcopenia in normal aging as well as in patients in various clinical settings.

## Results

### Affymetrix Global Gene Expression Analysis

In the present study we performed an expression profile analysis of mRNA deriving from human *soleus* biopsies taken two days before start, at base data collection-2 (pre = BDC-2) and at the end (head down tilt + 58 (HDT + 58)) of the bed rest (BR) to identify specific molecular pathways affected by RE and RVE countermeasures. Matched biopsies of *soleus* from pre- and end-bed rest individuals of the three different groups were analyzed using Affymetrix human Gene ST 1.0 array. Two way ANOVA statistical tests were used to identify genes significantly deregulated (p < 0.05) in end-BR versus pre-BR in each group. Filter criteria for each condition was adjusted to p < 0.05 and a fold change ± 1.3 was set, considering the fact, that the conditions (end vs. pre) were not so incisive compared to pathological settings.

### Magnitude of changes in global gene expression in CTR, RE and RVE groups following bed rest

**Figure 1:**
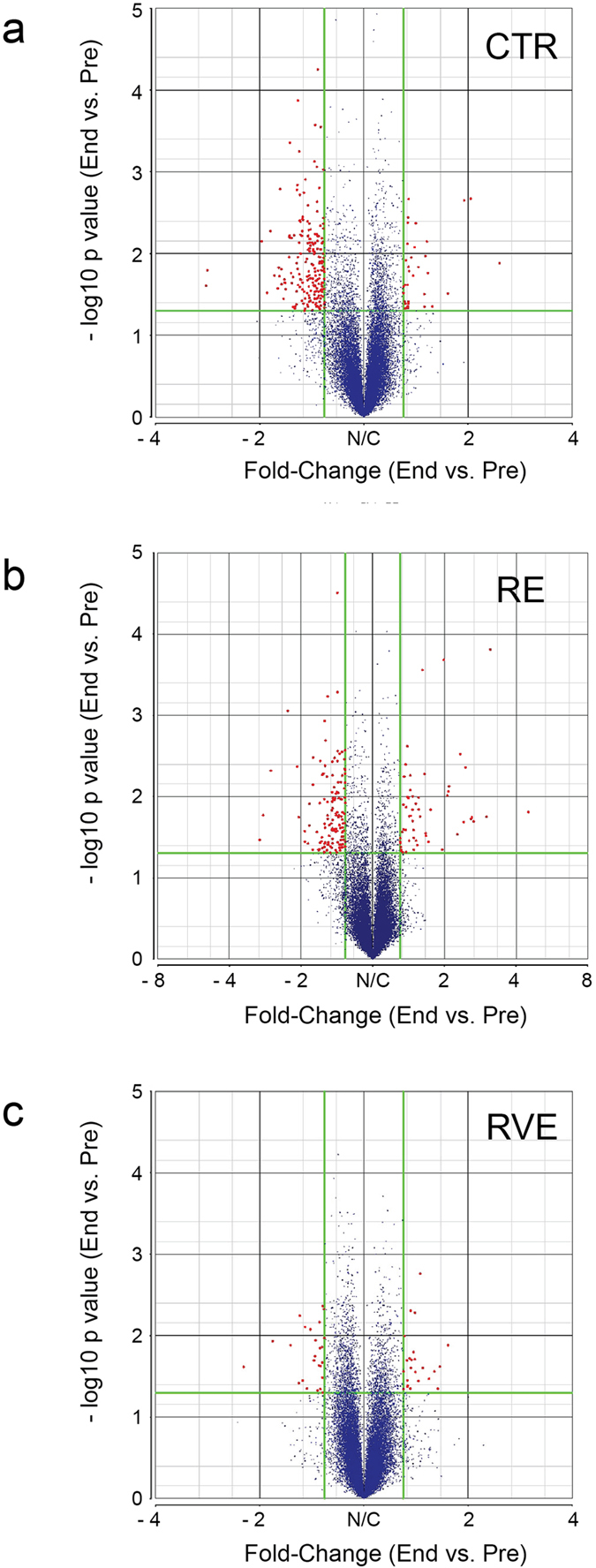
Effect of RE and RVE countermeasures on muscle-specific gene expression rearrangement after 60 days of bed rest. Volcano plots obtained by comparing end vs. pre skeletal muscle (SOL) biopsies (matched samples) in CTR, RE and RVE groups showing gene expression regulation in solei of bed rest subjects. Disuse-induced gene changes in CTR (**a**), RE (**b**) and RVE (**c**) groups given as single dots (red/blue color-coded) located within the area delineated by fold changes criteria. The different pattern of dots in each group reflect the differential gene profile outcome (red = significant change, blu = no change) for each intervention following long-term bed rest. Red dots represent significantly changed transcripts (p < 0.05) meeting < − 1.3 and >1.3 fold change cut off. Blue dots = changes below the cut off.

Volcano plots obtained by comparing end vs. pre conditions show that the CTR and RE groups are characterized by a similar magnitude of fold changes in the respective gene expression profile (Fig. 1a,b). By contrast, the volcano plot of the RVE group clearly shows a strongly reduced number of transcripts with fold change < − 1.3 and >1.3 (Fig. 1c). Moreover, we observed a high number of downregulated genes (< − 1.3 fold change, p < 0.05) ranging from −2.9 to −1.3 fold in the control group CTR (200 genes) and only 35 upregulated (>1.3 fold change, p < 0.05) genes ranging from 2.5 to 1.3 fold comparing end vs. pre condition. RE countermeasure reduced only in part this trend. In fact, in this group 140 genes were downregulated (ranging from −3 to −1.3 fold) and 66 genes upregulated (ranging from 4.5 to 1.3 fold). On the other hand, RVE countermeasure strongly counteracted disuse induced gene deregulation. Comparing end vs. pre in this group, we found 26 downregulated and 25 upregulated genes, respectively, ranging from −2.2 to −1.3 fold and from 1.7 to 1.3 fold.

### Relation of differentially expressed genes in each group

**Figure 2:**
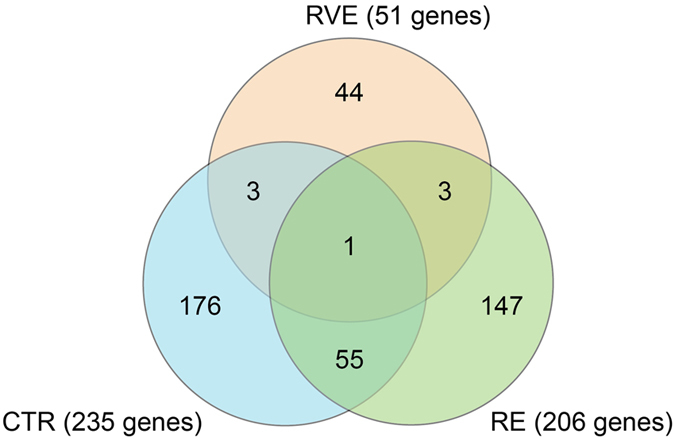
Venn diagram of genes differentially regulated in human soleus after 60 days of bed rest. Venn diagram showing the relation of genes differentially regulated comparing end vs. pre conditions in CTR, RE and RVE groups. The numbers of differentially regulated genes meeting p < 0.05 and < − 1.3 & >1.3 fold change criteria are shown for each group. The total number of transcripts differentially regulated in each group is given in brackets.

The Venn diagram (Fig. 2) shows the number of genes differentially regulated (fold change < − 1.3; >1.3; p < 0.05) in the three groups. Comparing the end vs. pre condition in the CTR and RE groups we observed a high number of differentially expressed genes, (235 in CTR vs. 206 in RE), while in the RVE group only 51 genes were differentially regulated (see supplementary table I-II-III for the complete lists of differentially regulated genes in CTR, RE and RVE groups). Moreover, we identified 56 common genes in CTR and RE groups, while only 4 genes revealed overlapping expression patterns in CTR vs. RVE groups. These results suggest that the outcome of RVE countermeasure is a close-to-baseline transcription profile compared to RE-induced gene expression rearrangement in human soleus following bed rest.

**Figure 3:**
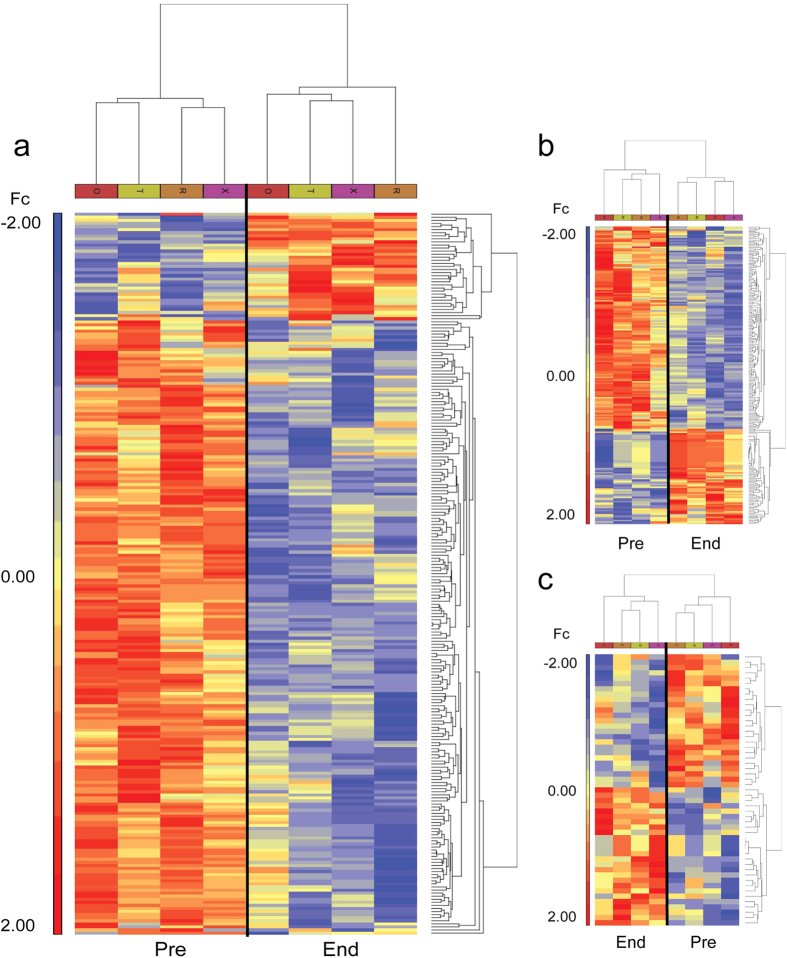
Hierarchical clustering of genes differentially expressed in skeletal muscle of CTR, RE and RVE groups. Expression profile of subject-matched solei muscle biopsies obtained by comparing end vs. pre bed rest in CTR (**a**), RE (**b**) and RVE (**c**) groups. The differentially regulated genes meeting p < 0.05 and < − 1.3 & >1.3 fold change criteria are included in the heat maps. The scale on the left of each graph represents normalized expression levels (blue, low expression; red, high expression). Fc = fold changes

Finally, the two way hierarchical clustering shows a partitioning of pre- and end-bed rest biopsies in all three groups (Fig. 3a–c), indicating that 60 days of bed rest induced significant alteration of gene expression that may not be completely prevented by both RE and RVE countermeasures. Although, RVE seems to counteract the bed rest-induced changes most efficiently.

### Efficacy of RVE countermeasure assessed by functional gene clustering analysis

**Figure 4:**
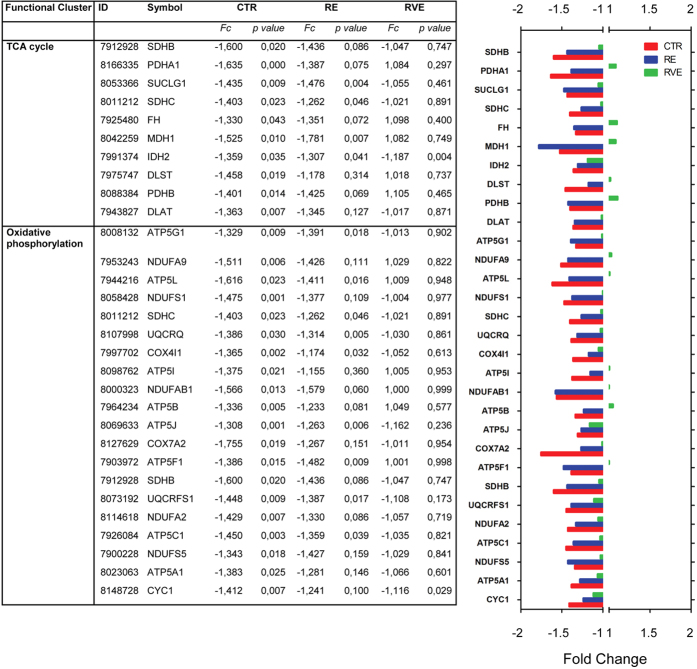
Differentially regulated genes linked to TCA cycle and oxidative phosphorylation in SOL after 60 days of bed rest in CTR group and efficacy of RE and RVE countermeasures. Table (left panel) lists functional gene clusters and bar chart (right panel) representing fold changes of differentially expressed gene transcripts (end vs. pre conditions) included in the main functional gene clusters in CTR (red), RE (blue) and RVE (green) groups. The bar chart highlights the outcome of the two exercise modalities (RE and RVE) vs. the control group (bed rest only) and reflects the efficacy of exercise as countermeasure on transcripts differentially regulated in the CTR group after bed rest. RVE (green bars) shows a close-to-baseline gene expression profile (less deviation from midline) vs. CTR (red) and RE (blue) in most of the differentially regulated functional genes listed here.

**Figure 5:**
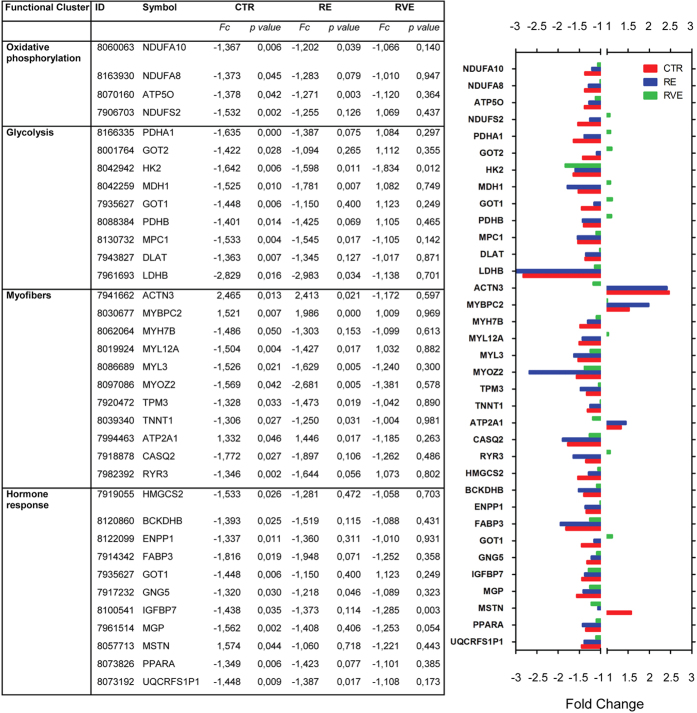
Differentially regulated genes linked to oxidative phosphorylation, glycolysis, myofibers and hormone response in SOL after 60 days of bed rest in CTR group and efficacy of RE and RVE countermeasures. Table (left panel) lists functional gene clusters and bar chart (right panel) representing fold changes of differentially expressed gene transcripts (end vs. pre conditions) included in the main functional gene clusters in CTR (red), RE (blue) and RVE (green) groups. The bar chart highlights the outcome of the two exercise modalities (RE and RVE) vs. the control group (bed rest only) and reflects the efficacy of exercise as countermeasure on transcripts differentially regulated in the CTR group after bed rest. RVE (green bars) shows a close-to-baseline gene expression profile (less deviation from midline) vs. CTR (red) and RE (blue) in most of the differentially regulated functional genes listed here.

**Figure 6:**
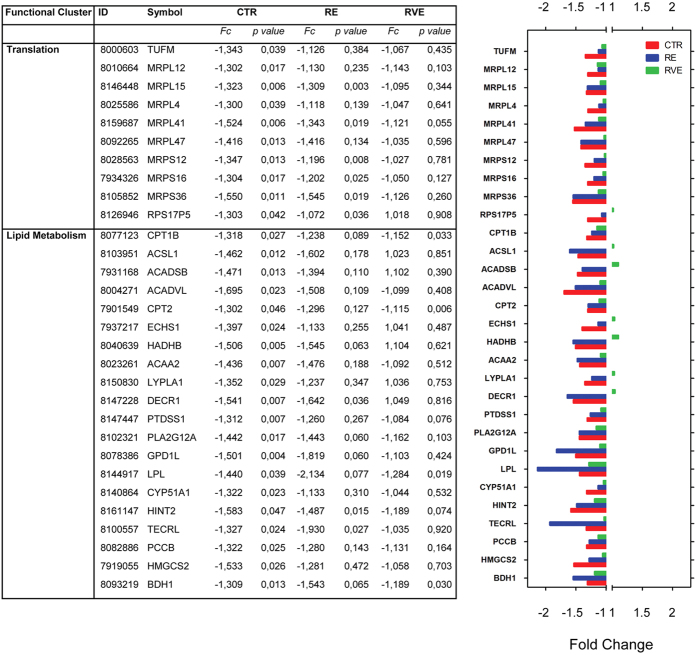
Differentially regulated genes linked to translation and lipid metabolism in SOL after 60 days of bed rest in CTR group and efficacy of RE and RVE countermeasures. Table (left panel) lists functional gene clusters and bar chart (right panel) representing fold changes of differentially expressed gene transcripts (end vs. pre conditions) included in the main functional gene clusters in CTR (red), RE (blue) and RVE (green) groups. The bar chart highlights the outcome of the two exercise modalities (RE and RVE) vs. the control group (bed rest only) and reflects the efficacy of exercise as countermeasure on transcripts differentially regulated in the CTR group after bed rest. RVE (green bars) shows a close-to-baseline gene expression profile (less deviation from midline) vs. CTR (red) and RE (blue) in most of the differentially regulated functional genes listed here.

To identify some of the molecular pathways specifically involved in disuse-induced skeletal muscle atrophy, functional gene clusters were obtained by using DAVID^18^ database. Since one of the aims of the study was to evaluate the efficacy of RE and RVE countermeasures in preventing gene deregulation observed in skeletal muscle after long-term bed rest, the pathway enrichment analysis was centred on the 235 end- vs. pre- differentially expressed genes in the control group (CTR). Seven different functional gene clusters were identified, four of which mainly linked to the mitochondrial functions of skeletal muscle fibers (see Figs 4, 5, 6). More specifically, glycolysis, tricarboxylic acid (TCA) cycle-, oxidative phosphorylation- and lipid metabolism-associated genes all undergo a significant downregulation. Particularly, in the TCA cycle cluster, we found a decreased expression of succinate dehydrogenase complex subunit C, of pyruvate dehydrogenase alpha 1 and beta, of fumarate hydratase and malate dehydrogenase 1. RE countermeasure weakly counteracted the effect of 60 days bed rest, particularly on TCA-related gene expression, whereas RVE strongly prevented disuse-induced deregulation of those genes. With respect to glycolysis, lactate dehydrogenase B and hexokinase 2 surprisingly were found to be downregulated in the CTR group, in this case, only RVE was able to abolish this trend. The larger functional cluster was composed by oxidative phosphorylation genes (24 genes), the most represented genes were subunits of ATP synthase complex, the cytochrome c1, the cytochrome c1 oxidase subunit and the ubiquinol-cytochrome c reductase. In this functional cluster RVE was able to prevent the downregulation of ATP synthase subunits. Moreover, as expected, a large number of genes involved in fatty acid metabolism (lipid metabolism) were found differentially regulated in the control group (Fig. 4c). Among those genes, enzymes crucial for fatty acid oxidation such as hydroxyacyl-CoA dehydrogenase and acetyl-CoA acyltransferase 2 or the monooxygenase cytochrome P450 (family 51, subfamily A, polypeptide 1) were found to be downregulated. Interestingly, in this functional cluster both RE and RVE countermeasures were able to prevent disuse-induced downregulation for most of the otherwise affected genes following prolonged disuse in bed rest.

Since mitoribosomes are crucial to orchestrate the translation of genes involved in ATP synthesis into the cell, different mitochondrial ribosomal proteins (MRPs) and the mitochondrial Tu translation elongation factor (TUFM), were found downregulated in skeletal muscle subjected to 60 days of bed rest. RE was able to prevent the differential regulation in the expression of only four of those genes (TUFM, MRPL12, MRPL4 and MRPL47), while RVE positively affected all those genes.

As expected, the functional cluster represented by structural and contractile proteins of myofibers reflected the typical myofiber slow to fast shift observed in disuse-induced atrophy. Comparing end vs. pre conditions in the control group, the expression of myosin binding protein C fast, the ATPase Ca^2+^ transporting fast twitch 1 and the actinin alpha 3 were increased whereas myosin light chain 3 and 12A, troponin T type 1, calsequestrin 2 and ryanodine receptor 3 were downregulated. Moreover, we found that transcripts linked to the regulation of skeletal muscle growth (hormone response cluster) were differentially regulated: Insulin-like growth factor binding protein 7 and pyrophosphatase/phosphodiesterase 1, a negative regulator of insulin receptor activation, were downregulated while myostatin, a well-known inhibitor of skeletal muscle differentiation and hypertrophy, was upregulated. As shown in Fig. 5, RE countermeasure counteracted only partially the effect of bed rest on the expression of these genes, whereas RVE mostly prevented disuse-induced gene deregulation. Interestingly, RE countermeasure appeared to be effective in rescuing the major part of genes grouped in the hormone response functional cluster, while RE did not affect the majority of genes coding for myofiber structural proteins.

RE countermeasure induced additional changes in gene expression not observed in CTR and RVE groups leading to a fast phenotype of skeletal muscle myofibers. As shown in Table 1 (muscle specific gene clusters exclusively regulated in RE group) myosin heavy chain 1, coding for the fast MyHC-2X, the perinatal myosin heavy chain 8, and different troponin fast-specific genes were all upregulated in RE end-condition. Intriguingly, two genes involved in skeletal muscle fibers type specification, Sox6 and prospero homeobox 1 (Prox1), were upregulated and downregulated respectively in the RE group after bed rest. Since Sox6 is known to be a repressor of slow isoform genes, upregulation is expected to induce a reduction in slow fibers specific genes exactly what we observed in the RE group. By contrast, Prox1 is known to play a crucial role as repressor of fast type skeletal muscle genes. We found Prox1 significantly downregulated in the RE group. In addition, four genes were identified as differentially regulated in both RE and RVE groups (end vs. pre): hexokinase (differentially regulated also in the CTR group), 24-dehydrocholesterol reductase, DENN/MADD contain domain 2C and methyltransferase like 21E pseudogene.

With respect to the genes differentially regulated in the RVE group, the small number (51 genes) did not allow for pathway enrichment analysis. The main functional clusters generated by means of DAVID analysis were: negative regulator of cell differentiation and proliferation, and catabolic processes (Table 2).

### Validation of microarray data by Proteomic analysis

Proteomic mass spectrometry analysis has become a reliable and powerful tool to study small changes in protein expression in a given sample in addition to posttranslational modifications.

Table 3 and Fig. 1S include only genes that have been “validated” by a proteomic approach (2D-DIGE and/or western blot analysis); in particular we selected 13 proteins listed in the 6 major metabolic pathways,(i.e. glycolysis, the TCA cycle, the malate shuttle, oxidative phosphorylation, translation and lipid metabolism). Ten of the thirteen proteins in end-CTR showed a different expression compared to RNA level; only 3 proteins (fumarate hydratase (FH), NADH dehydrogenase [ubiquinone] iron-sulfur protein 1 (NDUFS1), peroxisome proliferator-activated receptor alpha (PPARα)) were comparable with transcript levels. With regard to the comparison end- vs. pre-BR in RE, we detected a group of genes and proteins from microarray, 2D-DIGE, and western blot analysis that matched almost perfectly genes listed in Table 3. Finally comparing RVE-end vs. pre we observed that most of the transcripts and respective proteins were unchanged (p ≥ 0.05), although few specific protein isoforms analyzed by 2D-DIGE were differentially regulated. Nevertheless, the fold changes of these protein isoforms were always below ± 1.15 suggesting considerable differences between RE and RVE countermeasure protocol.

## Discussion

The main objectives of this study were (i) to investigate large-scale changes in the gene expression profile of a human antigravity postural muscle, the calf soleus, after 60 days of bed rest disuse and (ii) to assess the efficacy of two different countermeasures, i.e. resistive exercise without (RE) and with superimposed vibration mechanical stimulations (RVE) on global gene expression following long-term bed rest (BBR2–2^16^).

Pathway enrichment analysis in the CTR group after bed rest revealed a number of specific genes characterized by a general reduction in mRNAs encoding for enzymes associated with energy sources, such as anaerobic glycolysis, oxidative phosphorylation and the TCA cycle. In addition, lipid metabolism and several sarcomere and mitochondrial ribosomal protein transcripts (MRPs) were altered compared to baseline (at bed rest start).

In general, some of our findings correlated well with previous studies suggesting that early disuse adaptation events in human SOL in bed rest are mostly reflected by changes at the level of metabolic pathways and their relative switch toward a faster muscle phenotype in the absence of appropriate exercise countermeasures^1^,^19^.

In the present study, the application of a short duration conventional resistive exercise (RE) protocol as countermeasure was found to only partially counteract gene expression changes observed in the CTR group with some exceptions (Figs 4, 5, 6). By contrast, vibration mechanosignals superimposed on resistive loads (RVE) were able to almost fully counteract similar changes incurred during prolonged bed rest only (CTR) or with RE as exercise countermeasure, suggesting that RVE is a more effective countermeasure protocol than RE in preserving a close-to-baseline (pre-BR condition) gene expression. The present microarray data are complementary to previously published results obtained by morphological and biochemical analysis in the same muscle biopsies^7^,^8^. For example, the amount of both, fast-type and hybrid myofibers (co-expressing slow- and fast-type MyHCs) was significantly lower following RVE compared to the RE countermeasure protocol^8^.

The identified differentially regulated gene transcripts can be classified into several functional groups. The largest represents genes encoding for mitochondrial proteins involved in oxidative phosphorylation, glycolysis as well as in lipid metabolism that are all characterized by a transcriptional down-regulation following disuse in bed rest. Some of our results well correlate with previous reports suggesting that, chronic muscle disuse decreases mitochondrial activity^20^,^21^, and content as well as muscle oxidative capacity^12^,^14^,^22^. In addition to the decrease of cytochrome c mRNA, a decrease of the enzymatic activity of cytochrome c oxidase, succinate dehydrogenase, citrate synthase, and malate dehydrogenase was previously reported in rat muscle^23^,^24^,^25^. This results in a reduced capacity of the muscle to generate ATP aerobically: disused muscle becomes progressively more dependent on the glycolytic pathway^26^ although there are studies reporting that fast-type are more vulnerable than slow-type myofibers under a variety of atrophic conditions including disuse^27^. Interestingly, during chronic muscle disuse there is a loss in subsarcolemmal mitochondria^28^ in favour of a subpopulation more susceptible to pro-apoptotic stimuli such as the intermyofibrillar mitochondrial subpopulation^29^,^30^, a finding which may add a further notch to this complex adaptation program. By and large, it may be concluded that apoptotic susceptibility increases with muscle disuse.

Alterations of the mitochondrial respiratory chain activity are linked to free radical generation in human skeletal muscle^31^. At least 5% of the respired oxygen is converted into free radical superoxide (O_2._) which might further increase under particular pathological conditions such as, for instance, ischemia and reperfusion (for review^31^).

Our findings well correlate with previous observations suggesting that during chronic muscle disuse the mitochondrial respiratory chain activity is reduced which might result in an increased “oxidative stress” which, in turn, in absence of an increased antioxidant defence response, may likely contribute to muscle atrophy^32^,^33^.

Thus, the current understanding is that chronic muscle disuse may be characterized by a switch toward a glycolytic pathway taking place as the result of an increased mitochondrial dysfunction in oxidative myofibers that are basically more susceptible to pro-apoptotic stimuli, increased oxidative stress and altered calcium homeostasis all of which are well known as key signatures of disuse muscle atrophy.

Similarly, physical inactivity appears to impair lipid metabolism and increased fat storage after 20 days of bed rest^34^,^35^. Interestingly, the disuse-induced changes we determined by the present gene profiling in bed rest in the end-CTR group at the mitochondrial bioenergetics and lipid metabolism pathways were mostly prevented by RVE, and to a lesser extent, by the RE countermeasure protocols.

Further affected functions are represented by gene transcripts related to the myofiber itself (encoding for structural and signalling proteins), but also to several components of the hormone response and translation. With respect to genes coding for proteins related to the contractile apparatus, we noticed an increase in alpha-actinin-3, in myosin binding protein C fast type, and in sarcoplasmic/endoplasmic reticulum calcium ATPase1. Since the expression of these contractile proteins is confined to fast-type myofibers, their increase after 60 days of bed rest well correlated with the myofiber phenotype transition toward a faster phenotype rather than with their increased transcriptional regulation. All other transcripts of the same functional group such as the remaining components of the contractile apparatus and of calcium homeostasis were decreased after the bed rest period. However, since most of these deregulated transcripts are exclusively expressed in slow-type myofibers, this reduction well correlated with the decrease of the slow-type myogenic program during chronic disuse. The RVE countermeasure protocol strongly prevents the transcriptional deregulation of these genes, supporting previous data from structural and proteomic analysis^8^.

By contrast, comparison analysis of deregulated gene transcripts related to the hormone response showed no substantial differences between the RE and RVE countermeasure protocol suggesting that both protocols were capable of preventing some of the principal changes observed during bed rest disuse in the CTR group without exercise countermeasures. Whereas RVE, and, to a lesser extent, RE prevented transcriptional deregulation of some genes listed in the translation functional cluster (for example, the small and the large mitochondrial ribosomal proteins). Also in this respect, RVE appears to be more effective than RE in supporting substrate utilization and degradation in disused normal skeletal muscle for energy production.

Proteomic validation data analysis confirmed the efficacy of RVE countermeasure although an uncoupling between transcription and translation within subject-matched samples (end vs. pre) has been found mainly in the CTR group. This evidence is not unexpected when considering that transcriptional and translational machineries as well as proteolysis processes are strongly deregulated in atrophic myofibers (for review^36^). One hypothetical explanation could be that the different experimental tools we used had different degrees of sensitivity. Alternatively, the discrepancy between the data obtained by microarray, proteomics and western blotting might well be due to different gene products (mRNA vs. protein), regulatory mechanisms (structural vs. signalling proteins), half-life of different gene products taken into account, and finally the presence of one or more splice variants of the same gene. One possible drawback of the microarray technology used here certainly is given by the fact that some posttranslational modifications at the protein level might not have been detectable at microarray levels.

Together with previous^8^,^9^ findings, the current mapping of global gene expression in normal healthy subjects raise the ultimate question about the functional principles underlying most of the molecular changes occurring in disused vs. active human skeletal muscle and the identification of the direct effects of vibration mechanosignals on skeletal muscle gene transcription regulation.

Vibration stimulation is based on frequency-controlled rapid stretch shortenings by large numbers of neuroreflectory contractions that might enhance muscle force and power^37^,^38^ in addition to muscle flexibility, balance and proprioception^39^. All muscle responses might be further interpreted as the result of a direct neuronal enhancement by motor unit recruitment during vibration exercise (for review^39^) as a function of involuntary muscle contractions. Further evidence supporting this hypothesis came from recent studies proposing increased muscle strength output following vibration stimulation^40^,^41^. The present data are remarkable considering the wide spectrum of application of vibration therapy, ranging from support to limb casting to treatment of pathological conditions affecting the musculo-skeletal locomotor system in the course of several myopathies.

Increased oxidative stress levels following extended disuse in bed rest are reflected by elevated and reduced s-nitrosylated protein levels that, however, were attenuated by RVE exercise protocols in the BBR2-2 Study^42^. These results are consistent with the present gene array results from the same pool of study biopsy samples. For example the elevated number of deregulated enzymes in the CTR group of the mitochondrial respiratory chain, which are responsible for proton electrochemical gradients, was prevented in the RVE group suggesting that the RVE protocol might be able to enhance protective mechanisms against oxidative stress following chronic muscle disuse. Due to the limited number of deregulated genes in the RVE group, however, it was not possible to proceed for pathway enrichment analysis. Nevertheless, from the shortlist of genes exclusively regulated by vibration stimulation in bed rest we found the PANK2 gene (encodes for the pantothenate kinase protein), a key regulatory enzyme involved in the biosynthesis of coenzyme A (CoA), involved in fatty acid metabolism^43^; and DENND2C (DENN/MADD domain containing 2C) gene, which encodes for a protein that requires small GTPases activation^44^ in order to be able to interfere with a number of physiological cell functions including the actin cytoskeleton organization^45^.

### Conclusive remarks

The present comprehensive microarray data set and identification of functional gene clusters support the notion that resistive vibration exercise has a real impact on the normal transcription of a large number of muscle specific genes in apparently healthy subjects following bed rest immobilization. Most of the identified transcripts and proteins in this study are involved in the control of a normal muscle phenotype and energy metabolic pathways which are fundamental requirements of plasticity and adaptation processes in terms of a balanced control of adverse gene expression patterns in skeletal muscle fibers in disuse that remain to be characterized in further studies.

We conclude that mechanical vibration stimulation superimposed to resistive exercise exerts physiological responses on global gene transcription profiles controlling some of the key maintenance mechanisms related to the normal structural and functional status in human skeletal muscle following extended periods of disuse. The finding may also be of some value for optimization of current and future countermeasure protocols to avoid immobilization-induced muscle atrophy in clinical settings, rehabilitation, aging, and to maintain a close-to-baseline muscle quality of crew members in future planned long-term space missions.

## Methods

### Bed Rest study

The 2^nd^ Berlin Bed Rest Study (BBR-2) was conducted at the Charité University Medicine Berlin, Campus Benjamin Franklin, Berlin, Germany between the years 2007–2008. The detailed protocols including the anthropometric data and the two exercise countermeasure protocols (RE, RVE) are described elsewhere^16^.

### Human volunteers

In the present study, twelve (n = 12) out of twenty-three (n = 23) healthy male subjects from the original 2^nd^ BBR-2 study^16^ were randomly selected for the present GeneChip microarray analysis.

### Bed Rest protocol and experimental design

For this investigation, the twelve subjects from the BBR-2 study were randomized into three different subgroups, i.e. a non-trained bed rest (BR) control group (CTR, n = 4), a BR resistive exercise group (RE, n = 4), and a BR group with resistive exercise superimposed by whole-body vibration stimulation generated by pressing both feet on a vibrating foot plate (RVE, n = 4) in a supine position during prescribed countermeasure session in bed rest. Exercise (RE or RVE) protocols were carried out in a 6° head-down-tilt (HDT) posture on the same custom-made Galileo Space trainer (Novotec Medical GmbH, Pforzheim, Germany) whereas subjects from the CTR- group (“bed rest only”) remained strictly in the HDT position without being trained during bed rest.

### Countermeasure protocol

The adopted RVE countermeasure protocol consisted of short series (5–10 min 3 × /wk.) of resistive exercise bouts (RE) superimposed with lower frequency-controlled vibration mechanosignal patterns (with approximately 1000 contracting cycles per minute) particularly addressing the human soleus (SOL) postural muscle via plantar stimulation in supine body position in bed rest. Briefly, the RVE group performed the same exercise conditions (loading, intensity and number of bouts) as the RE group except that whole-body vibration was applied. The following exercises were performed in both RE and RVE groups: bilateral squats exercise (from 10° to 90° knee flexion and back with four seconds each for the concentric and eccentric phases, ~75–80% of pre bed-rest maximum voluntary contraction).

In the RVE group however, the following bouts/frequencies were applied: a) bilateral squats, vibration frequency 24 Hz, amplitude 3.5–4 mm, peak acceleration ~8.7 *g* where *g* = 9.81 ms^−2^; b) single leg heel raises, ~1.3 times body-weight, vibration frequency 26 Hz, amplitude 3.5–4 mm, peak acceleration ~10.2 *g*; c) double leg heel raises, ~1.8 times body-weight; vibration frequency 26 Hz, amplitude 3.5–4 mm, peak acceleration ~10.2 *g*. For more details of the BBR-2 study exercise protocol see Belavý *et al.*^16^.

### Muscle Biopsy

In total, we analyzed twenty-four biopsies (n = 12 pre-BR, n = 12 end-BR) from twelve (n = 12) subjects from three different groups (CTR, RE and RVE, n = 4 each). In all subjects and groups, open muscle biopsies from the muscle *soleus* (SOL) were harvested two days before start (BDC-2, pre-BR) and two days before end of bed rest (HDT58, end-BR)^16^. Muscle biopsies were snap-frozen immediately in liquid nitrogen and stored at −80 °C until further use. The Charité Universitätsmedizin Berlin Ethics Committee approved this study in accordance to the World Medical Association Code of Ethics, Declaration of Helsinki (1964). All participants underwent signed informed consent briefings and were aware of the fact that they could withdraw from the study at any time. They were also informed that, particularly, for muscle and blood specimens, experimental investigations would be conducted at any later time points after the bed rest campaign.

### RNA extraction and sample target preparation

Total RNA was isolated from muscle *soleus* biopsies using an RNeasy micro Kit (Qiagen, Hilden, Germany). Frozen tissue samples were ground to a fine powder under liquid nitrogen using a mortar and pestle. After adding the lysing buffer, a homogeneous lysate was achieved by flowing it 10 times through a needle connected with a sterile plastic syringe. After centrifugation of the tissue lysates the supernatant was phenol/chloroform-extracted^46^ Single-step method of RNA isolation by acid guanidinium thiocyanate-phenol-chloroform extraction. The aqueous layer was transferred and mixed with an equal volume of 70% ethanol and total RNA was extracted using RNeasy spin columns according to the manufacturer’s protocol. RNA integrity was checked by analyzing on the 2100 Bioanalyzer (Agilent technologies, PA, USA). Gene ST stands for sense target. Sense strand cDNA was generated from total RNA (input: 40–120 ng) using the Ambion WT Expression Kit (Life Technolgy) according to the manufacturer’s protocol. Subsequently, this generated single-strand DNA was fragmented with a combination of uracil DNA glucosylase (UDG) and apurinic/apyrimidinic endonuclease 1 (APE1) and then labelled using the WT terminal labelling kit (Affymetrix Inc., Santa Clara, CA, USA). The biotin-labelled and fragmented ss-cDNA was added with a final concentration of 23 ng/μl to a hybridization cocktail for hybridization to the Human Gene 1.0ST Array, also containing 50 pM control oligonucleotide B2, eukaryotic hybridization controls (bioB, bioC, bioD, 1,5, 5; 25 pM respectively), according to the manufacturer’s protocol (Hybridization Wash and Stain Kit, Affymetrix). The hybridization were carried out in the Affymetrix hybridization oven 640 at 45 °C and 60 rpm for 16 hrs, the staining and washing, processed in the Fluidics Station 450 and the scanning on the GeneChipScanner 3000 G7 system as recommended by Affymetrix (Affymetrix Inc., Santa Clara, CA, USA).

### Microarray hybridization

cRNA was hybridized on a GeneChip “HuGene 1.0 ST v1” by Affymetrix, offering whole-transcript coverage. Each of the 28.869 genes is represented on the array by approximately 26 probes spread across the full length of the gene, thus providing a more complete and accurate picture of gene expression.

### Microarray data analysis and pathway analysis

24 microarrays were hybridized using samples from pre (BR-pre) and from end (BR-end) bed rest muscle biopsies, 4 individuals (n = 4) for each group (CTR, RE and RVE). For data analysis the cel files were imported to the Partek^®^ Genomics Suite^®^ 6.6 software (Partek, St. Louis, MO, USA). For normalization the Robust Multichip Average algorithm was used. The processing steps involved in the RMA method are: Background correction, quantile Probe normalization across all arrays of the experiments, Log2 transformation of all signal values and median polished Probe set summarization. To identify differentially expressed genes, Analysis of Variance (two-way ANOVA) for paired data (end vs. pre) using Method of Moments^47^ was applied. The post versus pre state-pairs were contrasted according to the Fisher’s least Significant Difference method^48^. Lists comprised genes with fold changes greater than 1.3 or less than −1.3 and unadjusted p values less than or equal to 0.05 in each group: CTR, RE and RVE. Annotation was performed according to the *HuGene*-*1_0*-*st* Probe set Annotations, Release 35.

Gene ontology and pathway analysis were performed using DAVID v6.7 (The Database for Annotation, Visualization and Integrated Discovery^18^). The significance was evaluated by means of a modified Fisher’s exact test (EASE score), enrichments with p-values < 0.05 were considered significant^18^.

### Validation of microarray data by Proteomics

#### Protein extraction

For two-dimensional differences in gel electrophoresis (2D-DIGE) analysis and immunoblot assay: an aliquot of each frozen muscle was suspended in lysis buffer (urea 7 M, thiourea 2 M, CHAPS 4%, Tris 30 mM, and PMSF 1 mM) and solubilised by sonication on ice. Proteins were selectively precipitated by PlusOne 2D-Clean-up kit (GE Healthcare) to remove non protein impurities and re-suspended in lysis buffer. Protein extract was adjusted to pH 8.5 by 1 M NaOH and sample concentrations were determined using PlusOne 2D-Quant kit (GE Healthcare).

#### 2D-DIGE

Two-dimensional difference in gel electrophoresis protein labelling, 2D separation and analysis (for CTR, RE and RVE; n = 6 healthy subjects per group; SOL biopsy pre- and end-BR) were performed as previously described. The paired Student’s t-test (P < 0.01) was adopted to determine protein spots significantly different between pre- and end-BR by group analyses Reference 8. Paired tests can be used because each data point, in one group, corresponds to a matching data point in the other group. False discovery rate was applied as multiple test correction in order to keep the overall error rate as low as possible.

#### Protein identification by mass spectrometry

Proteins were identified by peptide mass fingerprinting (PMF) utilizing a MALDI-ToF/ToF mass spectrometer (Ultraflex III; Bruker Daltonics, Bremen, Germany), as described previously. To confirm protein identification, a MS/MS spectrum was collected by Ultraflex III MALDI-ToF/ToF mass spectrometer. Mass tolerance was set at 30 ppm and 0.5 Da for peptide and MS/MS fragment ion respectively (For further information about protein identification see Supplementary Table IV).

#### Orthogonal validation by immunoblotting of arrays and 2D-DIGE

Protein extracts (50 μg) from samples analyzed by 2D-DIGE were loaded in triplicate and resolved on 5-12% polyacrylamide gels, according to protein molecular weight. Blots were incubated with rabbit or goat polyclonal primary antibodies (Santa Cruz Biotechnology except when differently stated) as follows: anti-NADH dehydrogenase (ubiquinone) Fe-S protein 1 (NDUFS1), 1:500; anti-peroxisome proliferator-activated receptor alpha (PPARα), 1:500; anti-ATP synthase 5A (ATP5A1), 1:500; 1:500; anti-isocitrate dehydrogenase 2 (IDH2), 1:500; anti-glutamic-oxaloacetic transaminase 2, (GOT2, Bioss Antibodies), 1:1000; anti-fumarate hydratase (FH), 1:500. After washing, membranes were incubated with anti-rabbit (GE Healthcare) or anti-goat (Santa Cruz Biotechnology) secondary antibodies conjugated with horseradish peroxidase. Signals were visualized by chemiluminescence using the ECL Plus detection kit. Image analysis (Image Quant TL; Molecular Dynamics, Sunnyvale, CA, USA) was performed and followed by statistical analysis (paired Student’s t test, P < 0.05). Data were normalized against the total amount of proteins stained by Sypro Ruby (Molecular Probes) (Supplementary Fig 1).

## Additional Information

**Accession codes:** (http://david.abcc.ncifcrf.gov/); (www.genome.jp/kegg/pathway.html).

**How to cite this article**: Salanova, M. *et al.* Vibration mechanosignals superimposed to resistive exercise result in baseline skeletal muscle transcriptome profiles following chronic disuse in bed rest. *Sci. Rep.* **5**, 17027; doi: 10.1038/srep17027 (2015).

## Supplementary Material

Supplementary Information

## Figures and Tables

**Table 1:** Muscle specific functional clusters of differentially regulated genes in SOL after 60 days bed rest exclusively in RE group.

Functional Cluster	ID	Gene Symbol	RE	
			*Fc*	*p value*
**Myofibers**	7929653	ANKRD2	−2,883	0,017
	8012726	MYH1	4,508	0,015
	8012663	MYH8	1,470	0,020
	8066590	TNNC2	1,674	0,030
	7937728	TNNI2	1,561	0,014
	7937749	TNNT3	1,356	0,006
**Muscle development**	7946757	SOX6	1,325	0,048
	8058857	IGFBP5	1,349	0,025
	7994804	MYLPF	1,538	0,040
	7909681	PROX1	−1,448	0,026

The differentially regulated genes meeting p < 0.05 and < − 1.3 & >1.3 fold change criteria are included in the table.

**Table 2:** Functional clusters of genes differentially regulated in SOL after 60 days bed rest in RVE group.

Functional Cluster	ID	Gene Symbol	RVE	
			*Fc*	*p value*
**Regulator of cell differentiation and proliferation**	8162940	ABCA1	1,373	0,020
	8110520	HMGB3P22	1,335	0,043
	7943984	ZBTB16	−1,378	0,011
	7916432	DHCR24	−1,426	0,008
	8164269	ENG	−1,384	0,018
**Catabolic processes**	8052382	FANCL	1,454	0,002
	8003953	PSMB6	−1,362	0,047

The differentially regulated genes meeting p < 0.05 and < − 1.3 & >1.3 fold change criteria are included in the table.

**Table 3:** Shortlist of differentially regulated genes in CTR, RE and RVE groups, validated by 2D-DIGE proteomics and/or Western blot analysis.

Pathway	ID	Symbol	Uniprot	CTR						RE						RVE					
				Affymetrix		2D-DIGE		WB		Affymetrix		2D-DIGE		WB		Affymetrix		2D-DIGE		WB	
				Fc	p	Fc	p	Fc	p	Fc	p	Fc	p	Fc	p	Fc	p	Fc	p	Fc	p
Glycolysis	7961693	LDHB	Q5U077	−2,829	0,016	1,270	0,017			−2,983	0,034	−1,540	0,001			−1,138	0,701	1,030	0.257		
	8088384	PDHB	PI 1177	−1,401	0,014	1,550	0,006			−1,425	0,069	−1,190	0,003			1.105	0,465	1,060	0,112		
Citrate cycle (TCA cycle)	7991374	IDH2		−1.359	0.035	1.900	0.007	1,279	0,105	−1,307	0,041	−1,160	0,003	−1.441	0,022	−1,187	0,004	−1,100	0,058	−1,134	0,058
		IDH2	P48735			1.250	0,024					−1,250	0,003					−1,000	0,326		
	7925480	FH		−1,330	0,043			−1,138	0.430	−1.351	0,072			−1,741	0,014	1.098	0,400			1,029	0,539
Malate shuttle	7935627	GOT1		−1,448	0,006					−1,150	0,400					1.123	0,249				
		GOT1	P17174			1,170	0,120					−1,060	0,043					−1,020	0,276		
		GOT1	P17174			1.070	0,209					−1.130	0,030					−1,040	0,148		
		GOT1	P17174			1,270	0,032					−1,280	0,002					1,050	0,049		
	8042259	MDH1		−1,525	0,010					1,781	0,007					1,082	0,749				
		MDH1	P40925			1,320	0,021					1,170	0,008					−1,060	0,148		
		MDH1	P40925			1,010	0,399					−1.1.30	0,028					−1,120	0,034		
		MDH1	P40925			1,970	0,003					−1,200	0,007					−1,040	0,074		
	8001764	GOT2		1,422	0,028	1,630	0.003	1.102	0,138	1,094	0,265	−1,060	0,060	−1,352	0,002	1,112	0,355	1.400	0,015	1.779	0,015
Oxidative phosphorylation	8058428	NDUFS1		−1,475	0,001			−1,207	0,366	−1,377	0,109			−1,453	0,038	−1.004	0,977			1,017	0,848
	8023063	ATP5A1	P25705	−1,383	0,025	1,220	0,083	−1,039	0,611	−1,281	0,146	−1,280	0,001	−1,328	0,021	−1,066	0.601	−1,010	0,297	−1,126	0,427
	7964234	ATP5B		1,336	0,005					−1,233	0,081					1,049	0,577				
		ATP5B	P06576			−1,160	0,123					−1,340	0,004					−1,150	0,032		
		ATP5B	P06576			1,400	0,015					−1,280	0,002					−1,090	0,047		
Translation	8000603	TUFM	P49411	−1,343	0,039	1,010	0,424			−1,126	0,384	−1,240	0,007			−1,067	0,435	−1,060	0,091		
Lipid metabolism		ACAD VL	P49748	−1,695		1.690	0.007			−1,423	0,077	−1,400	0.004	−1.472	0,018			−1.010	0.294		
	8073826	PPARA		−1,349	0,006			−1,143	0,009							−1.101	0,385			−1,185	0,124
