# Supplementary material for: Vibration mechanosignals superimposed to resistive exercise result in baseline skeletal muscle transcriptome profiles following chronic disuse in bed rest
Source: Sci Rep. 2015 Nov 24;5:17027. doi: 10.1038/srep17027 (PMC4657004; doi:10.1038/srep17027)
Supplement: Supplementary Information [file srep17027-s1.pdf]

Vibration mechanosignals superimposed to resistive exercise result in baseline skeletal muscle transcriptome profiles following chronic disuse in bed rest

Michele Salanova*[1], Guido Gambara[1], Manuela Moriggi[2,3], Cecilia Gelfi[2,4,5], Michele Vasso[4], Ute Ungethuem[6], Daniel L. Belavý[7,8], Dieter Felsenberg[7], Paolo Cerretelli[4], and Dieter Blottner[1]

Supplemtary Table I

Column #	Transcript ID	Gene Symbol	Ref Seq	p-value	Fold-Change
16967	8024728	NMRK2	NM_170678	0,0246642	-2,86186
10766	7961693	LDHB	AY189689	0,0159462	-2,82906
10472	7958724	LOC100131138	NR_036513	0,00713504	-1,97745
12724	7981427	CKB	NM_001823	0,0301953	-1,90562
5068	7905131	CA14	AB025904	0,00532827	-1,86146
5965	7914342	FABP3	NM_004102	0,0185251	-1,81553
6389	7918878	CASQ2	BC022288	0,0266816	-1,77241
5820	7912670	UQCRHL	NM_001089591	0,0148629	-1,76429
27394	8127629	COX7A2	NR_029466	0,0189304	-1,75457
25196	8106689	CKMT2	NM_001825	0,00163374	-1,74525
20458	8059852	MSL3P1	AK301707	0,0155951	-1,73205
9851	7952335	SNORD14E	ENST00000364009	0,0238346	-1,7318
22506	8080184	ALAS1	AK300993	0,035871	-1,70666
16386	8019357	DCXR	AF113123	0,0207502	-1,6978
14951	8004271	ACADVL	AK097243	0,0226549	-1,69515
11048	7964832	RP11-254B13.3	OTTHUMT00000403191	0,0227763	-1,68685
31713	8168291	ITGB1BP2	AF110225	0,0274002	-1,68261
6897	7923824	SLC41A1	NM_173854	0,0110447	-1,64564
24281	8098084	ETFDH	BC011890	0,00627882	-1,64489
18810	8042942	HK2	BC064369	0,00603673	-1,64202
14095	7995362	GPT2	AY029173	0,00631515	-1,63887
31492	8166335	PDHA1	AK296341	0,00044006	-1,63478
23183	8087224	SLC25A20	AK301744	0,0172951	-1,62242
4414	7898663	PINK1	AB053323	0,00644916	-1,61903
8955	7944216	ATP5L	BC015128	0,0229572	-1,6162
12944	7982878	CHP1	BC031293	0,0452523	-1,60039
5847	7912928	SDHB	BC007840	0,0195422	-1,60014
10257	7956242	COQ10A	AY358861	0,00597803	-1,59195
30943	8161147	HINT2	BC033538	0,0470221	-1,58346

25190	8106573	THBS4	BC050456	0,0448394	-1,57914
24170	8097086	MYOZ2	NM_016599	0,0421324	-1,56853
14553	8000323	NDUFAB1	AF087660	0,0131119	-1,56617
10748	7961514	MGP	ENST00000539261	0,00165752	-1,56151
32138	8171823	APOO	AY359114	0,00145127	-1,55523
29815	8150204	RP1-273G13.2	OTTHUMT00000376526	0,0154114	-1,55311
25123	8105852	MRPS36	NM_033281	0,0110719	-1,55016
11666	7971294	FABP3P2	ENST00000458199	0,00013457	-1,54914
28585	8139128	TARP	BC030554	0,0242447	-1,54909
29458	8147228	DECR1	AK300069	0,00738212	-1,54067
14395	7998637	MSRB1	NM_016332	0,00056743	-1,53857
9494	7948910	SNHG1	AK095849	0,0179538	-1,53828
27683	8130732	MPC1	AF125101	0,00394566	-1,53345
6410	7919055	HMGCS2	BC044217	0,0257063	-1,53255
5236	7906703	NDUFS2	BC008868	0,00191008	-1,53232
23155	8086689	MYL3	BC009790	0,0207671	-1,52593
31016	8161558	AQP7P1	NR_002817	0,00408752	-1,52557
18741	8042259	MDH1	BC001484	0,0100242	-1,52477
30796	8159687	MRPL41	BC040035	0,00590533	-1,52407
21396	8069026	C21orf33	BC002370	0,0059316	-1,52245
28999	8143028	CHCHD3	AK310236	0,0210169	-1,51575
13084	7984405	C15orf61	AK294012	0,0379268	-1,51112
9947	7953243	NDUFA9	AF050641	0,00606353	-1,51102
18607	8040639	HADHB	AF113209	0,00531892	-1,50629
28044	8133876	CD36	L06850	0,0277036	-1,50424
16463	8019924	MYL12A	BC031972	0,00377565	-1,5041
13623	7990566	ETFA	AK292979	0,0225943	-1,50351
22346	8078386	GPD1L	BC028726	0,00358094	-1,50125
28748	8140371	TMEM120A	BC029487	0,00385435	-1,49809
13177	7985268	FAH	M55150	0,0408308	-1,49261
4324	7897824	MFN2	AF036536	0,0294277	-1,48941
20677	8062064	MYH7B	BC151242	0,0498478	-1,48553
19832	8053059	BOLA3	NM_212552	0,0355501	-1,47808
15661	8011747	SLC25A11	NM_003562	0,04749	-1,4778
20329	8058428	NDUFS1	BC030833	0,00124366	-1,47523
7621	7931168	ACADSB	NM_001609	0,0127124	-1,47139
24928	8103951	ACSL1	AK292798	0,0116675	-1,4624
16343	8018922	CYTH1	BC038385	0,0304966	-1,45866
12141	7975747	DLST	NM_001933	0,0192189	-1,45807
28518	8138640	CYCS	NM_018947	0,0424093	-1,45652
10649	7960716	PHB2	AF126021	0,0211156	-1,45438
30381	8155442	AQP7P1	ENST00000334576	0,00405768	-1,45382
26812	8121916	RSPO3	BC022367	0,00743406	-1,45153
7136	7926084	ATP5C1	BC000931	0,00256733	-1,45
19635	8051012	PREB	BC036185	0,0446956	-1,4492
14156	7996041	COQ9	BC054340	0,02083	-1,44851
6874	7923582	RP11-450D21.1	OTTHUMT00000087883	0,00602129	-1,44834

8057	7935627	GOT1	M37400	0,00618712	-1,44762
21831	8073192	UQCRFS1P1	ENST00000435169	0,00903399	-1,44759
32431	8174509	GNG5P2	ENST00000372054	0,00515665	-1,44482
12572	7979916	SYNJ2BP	AY534623	0,0254415	-1,44248
24729	8102321	PLA2G12A	AY359024	0,0165844	-1,4424
28320	8136849	GSTK1	NM_001143679	0,0324366	-1,44151
29226	8144917	LPL	M15856	0,0391705	-1,4404
31004	8161488	AQP7P1	ENST00000334576	0,00430795	-1,43952
24551	8100541	IGFBP7	BC017201	0,0348096	-1,43757
16819	8023261	ACAA2	NM_006111	0,00668613	-1,43563
25058	8105153	NNT	NM_012343	0,00938911	-1,43527
19864	8053366	SUCLG1	NM_003849	0,00869085	-1,43491
27465	8128383	COQ3	AK056955	0,00731302	-1,43326
26029	8114618	NDUFA2	AF077029	0,00667018	-1,42865
26483	8118667	MYL8P	ENST00000416347	0,00507309	-1,42555
11246	7967030	RNU4-1	NR_003925	0,020026	-1,42553
10592	7960117	PXMP2	AF250136	0,037771	-1,42532
13541	7989611	FAM96A	NM_032231	0,00797901	-1,42493
14707	8001764	GOT2	BC000525	0,0284554	-1,42205
23677	8092265	MRPL47	NM_020409	0,0134807	-1,41632
29614	8148728	CYC1	AK225796	0,00738322	-1,41156
7388	7928589	PPIF	NM_005729	0,0489536	-1,40765
12728	7981481	C14orf2	AF054175	0,0203256	-1,40465
15619	8011212	AC016292.3	OTTHUMT00000255248	0,0225839	-1,40339
7539	7930181	AS3MT	AF226730	0,0395195	-1,40183
23278	8088384	PDHB	BC000439	0,0137579	-1,40111
24923	8103904	LOC391722	XM_003960344	0,008905	-1,39879
14821	8002987	CMC2	AF201935	0,00940551	-1,39853
8637	7940904	VEGFB	BC008818	0,00777193	-1,39828
8193	7937217	ECHS1	ENST00000368547	0,0244065	-1,39707
30406	8155554	AQP7P4	ENST00000447083	0,00952074	-1,39521
26673	8120860	BCKDHB	NM_183050	0,025001	-1,39282
22694	8082066	FAM162A	AF201944	0,035622	-1,38788
4934	7903972	ATP5F1	NM_001688	0,015155	-1,38649
25331	8107998	UQCRQ	NM_014402	0,029525	-1,38612
18907	8043564	FAHD2A	AF151863	0,00580322	-1,3839
16795	8023063	ATP5A1	AK289457	0,0250135	-1,38344
6345	7918437	LAMTOR5	BC062619	0,0248115	-1,3828
25300	8107673	GRAMD3	AK024966	0,00741145	-1,3822
7934	7934255	MICU1	BC004190	0,0166765	-1,38139
26588	8119898	VEGFA	NM_001025366	0,00026713	-1,38117
30816	8159945	AK3	NM_016282	0,0186034	-1,37852
21509	8070160	ATP5O	NM_001697	0,0421796	-1,37758
24364	8098762	ATP5I	D50371	0,0214755	-1,37526
19869	8053406	RETSAT	AY358568	0,0402995	-1,37389
31247	8163930	NDUFA8	NM_014222	0,0447634	-1,37337
7549	7930304	GSTO1	BX537431	0,0288593	-1,37253

4365	7898249	SLC25A34	BC027998	0,0358314	-1,3714
15395	8008664	AKAP1	ENST00000481416	0,0264879	-1,36869
24339	8098576	SLC25A4	AB209764	0,00086971	-1,36861
15474	8009476	MAP2K6	AK290747	0,0435829	-1,36608
14303	7997702	COX4I1	ENST00000568339	0,00233043	-1,36549
26774	8121601	FAM26E	NM_153711	0,0210064	-1,36486
19881	8053533	IMMT	AY232292	0,00306999	-1,36485
8919	7943827	DLAT	BC039084	0,00703372	-1,36288
22674	8081867	TIMMDC1	AL390090	0,00880592	-1,36184
13715	7991374	IDH2	BC009244	0,0346737	-1,35935
15768	8013015	CENPV	ENST00000476243	0,00617272	-1,35909
26758	8121502	GTF3C6	AF361492	5,60E-05	-1,35746
25758	8112331	ISCA1	AF284752	0,0162069	-1,35682
18375	8038792	ETFB	AF370381	0,0294024	-1,3523
29872	8150830	LYPLA1	AF081281	0,0292877	-1,35159
32886	8178188	RNF5	BC004155	0,0328011	-1,3507
22011	8074939	CHCHD10	BC065232	0,0455097	-1,34958
4661	7901212	UQCRH	BC093060	0,0110186	-1,3493
21889	8073826	PPARA	NM_005036	0,00615201	-1,3491
11116	7965541	FGD6	NM_018351	0,0202965	-1,34796
17344	8028563	MRPS12	NM_021107	0,0131384	-1,34656
12903	7982392	RYR3	NM_001036	0,0015749	-1,34623
5099	7905481	HMGN3P1	ENST00000453847	0,0464188	-1,34593
14578	8000603	TUFM	BC001633	0,0390668	-1,34286
4581	7900228	NDUFS5	AF047434	0,0181612	-1,34255
7146	7926170	DHTKD1	NM_018706	0,0126405	-1,34192
28720	8140085	MLXIPL	AF156603	0,0479365	-1,34175
26824	8122099	ENPP1	BC059375	0,0110484	-1,33734
24895	8103722	HSP90AA6P	AY956762	0,00891512	-1,33627
10982	7964234	ATP5B	NM_001686	0,00456667	-1,33575
10270	7956401	SHMT2	AK055053	0,0355532	-1,33299
12132	7975661	COQ6	NM_182476	0,0277357	-1,33052
14890	8003667	SERPINF1	AF400442	0,00028268	-1,33039
8097	7936091	USMG5	NM_032747	0,0285548	-1,33008
7060	7925480	FH	U59309	0,043157	-1,33007
15352	8008132	ATP5G1	NM_005175	0,00897755	-1,32912
6584	7920472	TPM3	NM_152263	0,0326107	-1,32842
24556	8100557	TECRL	AL833108	0,0239235	-1,32737
14478	7999419	DEXI	BC037569	0,0130795	-1,32334
29370	8146448	MRPL15	AF161494	0,00649605	-1,32262
8071	7935810	NDUFB8	AL080056	0,00746892	-1,3221
22765	8082886	PCCB	AK295312	0,0252198	-1,32165
28790	8140864	CYP51A1	BC032322	0,0227197	-1,32157
14115	7995580	RP11-297L17.6	OTTHUMT00000422548	0,0123894	-1,32145
6234	7917232	GNG5	NM_005274	0,0295228	-1,31951
22237	8077123	CHKB-CPT1B	ENST00000492556	0,0274496	-1,31754
10984	7964248	SNORD59A	NR_002737	0,0144896	-1,31394

29476	8147447	PTDSS1	AK293036	0,00718653	-1,31246
30854	8160297	PLIN2	NR_038064	0,0334659	-1,3115
11253	7967072	COQ5	BX647562	0,0225298	-1,31094
32134	8171791	SMPX	AF129505	0,0160914	-1,31086
23770	8093219	BDH1	NM_004051	0,0126542	-1,30856
15445	8009176	TACO1	AK094052	0,0112603	-1,30803
29649	8149071	ANGPT2	AK290070	0,00367376	-1,308
21454	8069633	ATP5J	NM_001003701	0,00094001	-1,30795
6133	7916219	SELRC1	NM_023077	0,00432329	-1,30787
12595	7980098	ALDH6A1	AK311478	0,00493846	-1,30781
29832	8150356	RNF5P1	NR_003129	0,0151118	-1,30657
18444	8039340	TNNT1	AK291352	0,0271557	-1,30625
7944	7934326	MRPS16	NM_016065	0,0165686	-1,30428
11478	7969428	UCHL3	BC018125	0,0204526	-1,30296
27335	8126946	RPS17P5	ENST00000498293	0,0418599	-1,3025
15571	8010664	MRPL12	BC002344	0,0169397	-1,30198
4703	7901549	CPT2	U09648	0,0463054	-1,30174
17047	8025586	MRPL4	NM_146388	0,0393747	-1,3003
26252	8116807	SNRNP48	NM_152551	0,0306412	1,3017
31776	8168687	RNA5SP510	ENST00000364697	0,0385056	1,30901
8285	7938002	OR51J1	ENST00000332043	0,0242718	1,31561
26657	8120654	KCNQ5	NM_019842	0,0469015	1,31708
29543	8148059	DEPTOR	BC012040	0,0120725	1,32308
14006	7994463	ATP2A1	AK291314	0,0460441	1,33175
25719	8111998	HCN1	AF488549	0,0452584	1,33297
20384	8058997	PRKAG3	NM_017431	0,00763516	1,33466
18855	8043276	SNORD94	NR_004378	0,0241659	1,33657
13497	7989069	PYGO1	AF457207	0,00419197	1,33955
25072	8105323	HSPB3	NM_006308	0,0145763	1,34454
16491	8020179	CHMP1B	BC065933	0,0409665	1,34492
22610	8081241	CMSS1	BC006475	0,0111852	1,37083
24907	8103812	ASB5	NM_080874	0,0186487	1,38841
30469	8156164	KIF27	AY237536	0,00839008	1,39908
9514	7949075	TRMT112	AF161501	0,00425932	1,41301
28211	8135804	ASB15	NM_080928	0,0146302	1,43768
29362	8146393	C8orf22	NM_001256598	0,0315947	1,4939
22843	8083616	MLF1	BX647666	0,045008	1,495
20639	8061605	MYLK2	BC092413	0,0107687	1,50608
17540	8030677	MYBPC2	BC130536	0,00715419	1,5211
22957	8084766	TP63	BC039815	0,0173118	1,53009
20269	8057713	MSTN	AF104922	0,0444873	1,5744
12842	7982070	SNORD115-32	NR_003347	0,00223595	1,94835
19187	8046078	B3GALT1	NM_020981	0,00214072	2,03444
8700	7941662	ACTN3	AK125851	0,013114	2,46534

Supplemtary Table II

Column #	Transcript ID	Gene Symbol	Ref Seq	p-value	Fold-Change
10766	7961693	LDHB	AY189689	0,0341547	-2,98323
7490	7929653	ANKRD2	AJ583444	0,0168741	-2,88326
24170	8097086	MYOZ2	NM_016599	0,00479207	-2,68061
11531	7969955	METTL21EP	NR_026965	0,000875796	-2,27212
31745	8168470	COX7B	BC018386	0,0042611	-2,0766
10472	7958724	LOC10013113 8	NR_036513	0,0179003	-2,04673
24556	8100557	TECRL	AL833108	0,0265448	-1,92967
26812	8121916	RSPO3	BC022367	0,0359563	-1,92582
12724	7981427	CKB	NM_001823	0,0379632	-1,87084
14095	7995362	GPT2	AY029173	0,0228314	-1,84782
6155	7916432	DHCR24	AF261758	0,0122073	-1,8449
30558	8157027	NIPSNAP3B	BC017914	0,0450978	-1,78626
18741	8042259	MDH1	BC001484	0,00716908	-1,78065
30260	8154367	TYRP1	BC052608	0,00331317	-1,7761
31798	8168873	ARMCX6	AK097018	0,0272228	-1,71831
20711	8062461	LBP	NM_004139	0,0449267	-1,69005
6897	7923824	SLC41A1	NM_173854	0,0462943	-1,68643
11576	7970473	XPO4	NM_022459	0,0444792	-1,67573
8822	7942889	TMEM126A	BC007875	0,0120563	-1,66995
20198	8056693	FASTKD1	NM_024622	0,0371339	-1,66172
28518	8138640	CYCS	NM_018947	0,00363998	-1,65966
29458	8147228	DECR1	AK300069	0,0356331	-1,64235
14821	8002987	CMC2	AF201935	0,031745	-1,63465
13623	7990566	ETFA	AK292979	0,043348	-1,63139
23155	8086689	MYL3	BC009790	0,00539012	-1,62853
5820	7912670	UQCRHL	NM_001089591	0,00518419	-1,60805
11048	7964832	RP11-254B13.3	OTTHUMT0000040319 1	0,0481014	-1,60275
18810	8042942	HK2	BC064369	0,0107423	-1,59773
28183	8135464	DLD	AK294146	0,0417385	-1,59771
31713	8168291	ITGB1BP2	AF110225	0,0483537	-1,59588
23129	8086451	HIGD1A	AF077034	0,0216688	-1,59572
28553	8138912	LSM5	NM_012322	0,0380134	-1,59541
28767	8140534	SEMA3C	AB000220	0,0215003	-1,59342
30514	8156571	C9orf3	AF043897	0,00116394	-1,59327
19637	8051028	RNY4P15	ENST00000365142	0,0242452	-1,58618
10837	7962516	SLC38A1	BX647547	0,00416515	-1,58558
29815	8150204	RP1-273G13.2	OTTHUMT0000037652 6	0,00203504	-1,57906
7357	7928318	MCU	BC034235	0,0456902	-1,56028
19818	8052940	PAIP2B	NM_020459	0,0056535	-1,55979
28962	8142663	NDUFA5	ENST00000355749	0,0462105	-1,55966

30900	8160663	AQP7	AB006190	0,0116567	-1,55005
15474	8009476	MAP2K6	AK290747	0,0388497	-1,54875
25123	8105852	MRPS36	NM_033281	0,0187974	-1,54506
27683	8130732	MPC1	AF125101	0,0171085	-1,54492
11464	7969339	RNA5SP30	ENST00000365552	0,000583698	-1,54457
5465	7909285	PFKFB2	NM_006212	0,0365918	-1,53614
4661	7901212	UQCRH	BC093060	0,0390456	-1,53172
25039	8104930	SLC1A3	BC037310	0,0305091	-1,52707
28588	8139160	FAM183B	AK124132	0,0305459	-1,52134
22305	8078008	LSM3	AF182289	0,049303	-1,50703
10075	7954460	LYRM5	BC071769	0,0411581	-1,50243
28999	8143028	CHCHD3	AK310236	0,0215372	-1,49237
30943	8161147	HINT2	BC033538	0,0148508	-1,48671
13621	7990555	NRG4	CR749519	0,011501	-1,48293
4934	7903972	ATP5F1	NM_001688	0,00884293	-1,48221
11478	7969428	UCHL3	BC018125	0,0247151	-1,4768
18856	8043278	MRPL35	NM_016622	0,0377046	-1,47656
19864	8053366	SUCLG1	NM_003849	0,00371154	-1,47639
29244	8145136	PPP3CC	AK299415	0,00547274	-1,47605
10193	7955719	HIGD1AP1	ENST00000550979	0,0260425	-1,47604
26483	8118667	MYL8P	ENST00000416347	0,0329901	-1,47502
6584	7920472	TPM3	NM_152263	0,0193649	-1,47324
19747	8052250	MTIF2	AF494407	0,0139501	-1,47244
14395	7998637	MSRB1	NM_016332	0,010493	-1,46875
12728	7981481	C14orf2	AF054175	0,025579	-1,46633
30893	8160587	NDUFB6	NM_002493	0,0105541	-1,46341
21021	8065444	ACSS1	BC039261	0,0109249	-1,46219
20242	8057377	CCDC141	AK129847	0,00767851	-1,45456
10838	7962537	SLC38A2	AF298897	0,00334674	-1,4493
23426	8089785	POPDC2	BC026911	0,0110402	-1,44841
5498	7909681	PROX1	ENST00000498508	0,0262169	-1,44809
10257	7956242	COQ10A	AY358861	0,0105283	-1,43752
13612	7990436	COX5A	BC024240	0,0269932	-1,42953
30381	8155442	AQP7P1	ENST00000334576	0,0161192	-1,42756
16463	8019924	MYL12A	BC031972	0,0169813	-1,4271
31016	8161558	AQP7P1	NR_002817	0,00916767	-1,4268
11534	7969977	ATP5G1P5	ENST00000429024	0,0247888	-1,42444
31004	8161488	AQP7P1	ENST00000334576	0,0169709	-1,42125
25944	8113773	ALDH7A1	AK295526	0,0487648	-1,41848
11997	7974190	DNAJC19P9	ENST00000555084	0,00879656	-1,41407
8955	7944216	ATP5L	BC015128	0,0156372	-1,41145
21056	8065756	FDX1P1	ENST00000449115	0,0264145	-1,41
12631	7980485	DIO2	NM_001242502	0,000519864	-1,40685
32431	8174509	GNG5P2	ENST00000372054	3,09E-05	-1,4064
12038	7974483	KTN1	AY264265	0,00274676	-1,40219
19829	8053036	TPRKB	BC029492	0,0365757	-1,40164
12026	7974402	RP11-	OTTHUMT0000041195	0,00666747	-1,3953

		368P15.1	6		
23559	8091255	PAQR9	OTTHUMT0000035453 8	0,00905632	-1,39377
15352	8008132	ATP5G1	NM_005175	0,017909	-1,39112
26009	8114455	HSPA9	BC000478	0,0105134	-1,3883
21831	8073192	UQCRFS1P1	ENST00000435169	0,016894	-1,38663
16889	8023920	TXNL4A	ENST00000355491	0,0388199	-1,38379
25196	8106689	CKMT2	NM_001825	0,0222639	-1,38329
25069	8105311	NDUFS4	AF020351	0,0226405	-1,37984
8923	7943867	BCO2	NM_031938	0,04263	-1,37915
19832	8053059	BOLA3	NM_212552	0,0434574	-1,37834
26734	8121300	QRSL1	BC006084	0,0461946	-1,37042
7361	7928354	FAM149B1	AB023191	0,0244563	-1,36871
11363	7968270	ATP5EP2	NR_002162	0,0106155	-1,36471
7136	7926084	ATP5C1	BC000931	0,0394044	-1,35927
8438	7939411	C11orf74	AK290833	0,00666792	-1,35906
22469	8079733	KLHDC8B	BC039083	0,0343826	-1,35707
20556	8060839	CRLS1	NM_019095	0,0175606	-1,35456
25210	8106776	COX7C	AK026505	0,0107894	-1,35116
27655	8130499	DYNLT1	BC029412	0,00280172	-1,34797
20563	8060947	SNAP25-AS1	NR_040710	0,0422871	-1,3466
29926	8151223	SLCO5A1	AF205075	0,00551645	-1,34423
30115	8152750	TMEM65	BC041379	0,0276807	-1,3441
30796	8159687	MRPL41	BC040035	0,0191951	-1,34296
30406	8155554	AQP7P4	ENST00000447083	0,0421382	-1,34125
21738	8072274	UQCR10	NM_001003684	0,0240217	-1,34023
8097	7936091	USMG5	NM_032747	0,0156481	-1,33954
29036	8143433	MRPS33	AF151897	0,0385992	-1,33559
19897	8053662	RNY4P15	ENST00000365142	0,0213065	-1,33213
20130	8055672	MMADHC	NM_015702	0,0307393	-1,33113
21928	8074227	CECR5	AF273271	0,0265419	-1,33055
23235	8087860	TWF2	AF109365	0,0339143	-1,32584
22755	8082767	TMEM108	AY358381	0,00794446	-1,32131
19174	8045946	PSMD14	AK055128	0,0262809	-1,32078
10484	7958846	PTPN11	NM_002834	0,0161011	-1,31667
25331	8107998	UQCRQ	NM_014402	0,00453219	-1,31442
31156	8162880	MRPL50	BC032008	0,0212542	-1,30986
29370	8146448	MRPL15	AF161494	0,00269429	-1,30872
13715	7991374	IDH2	BC009244	0,0411835	-1,30727
12459	7978766	FKBP3	BC016288	0,00674744	-1,30723
10453	7958414	ISCU	AY009127	0,00519898	-1,30696
12132	7975661	COQ6	NM_182476	0,0118946	-1,30224
17585	8030991	TPM3P9	BX647962	0,0359583	-1,30092
16615	8021376	NEDD4L	NM_001144968	0,035824	1,30343
11903	7973306	ABHD4	NM_022060	0,0374757	1,30491
23323	8088848	PDZRN3	BC152417	0,0417614	1,31193
7582	7930714	ATRNL1	NM_207303	0,0451949	1,31694

9250	7946757	SOX6	NM_017508	0,0477499	1,32517
11599	7970676	SHISA2	NM_001007538	0,0272377	1,32868
20356	8058627	ERBB4	L07868	0,0486083	1,3384
20372	8058857	IGFBP5	NM_000599	0,0253051	1,34897
8237	7937749	TNNT3	NM_001042782	0,00565157	1,35561
13590	7990151	PKM	NM_182470	0,0258414	1,37628
12851	7982088	SNORD115-41	NR_003356	0,00402116	1,37736
20384	8058997	PRKAG3	NM_017431	0,00239248	1,3958
10133	7955078	PFKM	NM_001166686	0,010477	1,41356
8552	7940116	LOC399898	AK128188	0,0350596	1,42112
14933	8004086	USP6	AY143550	0,0174139	1,42198
30950	8161190	HMGB3P24	ENST00000433260	0,00550442	1,43684
27583	8129677	SGK1	ENST00000367858	0,00834546	1,43785
14006	7994463	ATP2A1	AK291314	0,0171774	1,44604
12827	7982040	SNORD115-17	NR_003309	0,0145054	1,46745
12828	7982042	SNORD115-17	NR_003309	0,0145054	1,46745
12829	7982044	SNORD115-17	NR_003309	0,0145054	1,46745
15737	8012663	MYH8	M36769	0,0199156	1,47001
4743	7901951	PGM1	AK294186	0,00993514	1,47061
6854	7923386	LMOD1	BC080187	0,025812	1,48625
27664	8130578	SNORA20	NR_002960	0,0452745	1,48955
12840	7982066	SNORD115-6	NR_003298	0,0324138	1,49003
12802	7981990	SNORD116@	AF241255	0,0122551	1,51802
12841	7982068	SNORD115-31	NR_003346	0,0349301	1,52165
14035	7994804	MYLPF	AF363061	0,0397657	1,53773
22389	8078918	RPSA	AK055991	0,0102669	1,55927
8235	7937728	TNNI2	BC032148	0,014403	1,56085
6379	7918768	DENND2C	NM_001256404	0,0175187	1,58184
31117	8162492	FBP2	NM_003837	0,000275962	1,61656
12848	7982082	SNORD115-38	NR_003353	0,00524907	1,64956
21146	8066590	TNNC2	NM_003279	0,0304911	1,67429
12843	7982072	SNORD115-33	NR_003348	0,0360868	1,71829
12817	7982020	SNRPN	AF400492	0,0144999	1,75172
12833	7982052	PAR4	AF019617	0,0449535	1,95849
17540	8030677	MYBPC2	BC130536	0,000207743	1,98636
12816	7982018	SNORD115-6	NR_003298	0,00970129	2,05688
12811	7982008	SNRPN	AF400495	0,00863166	2,08075
12823	7982032	SNRPN	AF400495	0,00863166	2,08075
12826	7982038	SNRPN	AF400495	0,00863166	2,08075
12852	7982090	SNORD115-6	NR_003298	0,00745348	2,09804
12830	7982046	SNORD115-20	NR_003312	0,0290965	2,26568
12835	7982056	SNORD115-25	NR_003342	0,0029895	2,32806
8700	7941662	ACTN3	AK125851	0,0205852	2,41325
12854	7982094	SNORD115-44	NR_003359	0,00438838	2,44972
12836	7982058	SNRPN	AF400501	0,0187137	2,58872
12815	7982016	SNRPN	AF400501	0,0185171	2,60413
12819	7982024	SNRPN	AF400501	0,0185171	2,60413

12822	7982030	SNRPN	AF400501	0,0185171	2,60413
12821	7982028	SNRPN	AF400501	0,0178829	2,60884
12839	7982064	SNRPN	AF400501	0,0178829	2,60884
12846	7982078	SNRPN	AF400501	0,0178829	2,60884
12853	7982092	SNRPN	AF400501	0,0178829	2,60884
12832	7982050	SNRPN	AF400501	0,02021	2,66139
12849	7982084	SNRPN	AF400501	0,017675	3,00755
12842	7982070	SNORD115-32	NR_003347	0,000155295	3,11621
15739	8012726	MYH1	NM_005963	0,0154771	4,50829

Supplemtary Table III

Column #	Transcript ID	Gene Symbol	Ref Seq	p-value	Fold-Change
11531	7969955	METTL21EP	NR_026965	0,024196	-2,22599
18810	8042942	HK2	BC064369	0,011741	-1,83401
18223	8037298	CD177	AF146747	0,013099	-1,62823
8944	7944082	TAGLN	NM_001001522	0,005685	-1,53247
7703	7932082	CCDC3	NM_031455	0,007828	-1,47895
6155	7916432	DHCR24	AF261758	0,008427	-1,42645
31284	8164269	ENG	NM_000118	0,017887	-1,38436
8934	7943984	ZBTB16	ENST00000335953	0,011447	-1,37828
14925	8003953	PSMB6	BC000835	0,047076	-1,36227
10236	7956013	BLOC1S1	BC066340	0,00683	-1,34241
10477	7958784	ALDH2	BC002967	0,014208	-1,33801
14776	8002403	MTSS1L	AB115770	0,045329	-1,33572
13084	7984405	C15orf61	AK294012	0,023663	-1,33322
16374	8019243	PPP1R27	NM_001007533	0,013845	-1,32028
15733	8012591	DHRS7C	BC147024	0,021293	-1,3193
17750	8032392	MKNK2	NM_199054	0,03255	-1,31764
23689	8092404	MAP6D1	NM_024871	0,04969	-1,31536
28748	8140371	TMEM120A	BC029487	0,004345	-1,31517
5696	7911371	C1orf170	NR_027693	0,004722	-1,30596
10906	7963348	KRT121P	ENST00000529785	0,010729	-1,30197
30469	8156164	KIF27	AY237536	0,0275	1,30256
4864	7903188	PTBP2	AB051232	0,010117	1,30919
8851	7943160	SCARNA9	NR_002569	0,020016	1,33061
25579	8110520	HMGB3P22	ENST00000451823	0,042557	1,33488
12914	7982535	C15orf41	AF114263	0,041253	1,33738
4886	7903425	AMY1A	BC132985	0,019019	1,35335
4887	7903440	AMY1A	BC132985	0,019019	1,35335
6320	7918134	AMY1A	BC063129	0,019019	1,35335
20433	8059648	RNY4P19	ENST00000362530	0,04474	1,362
4885	7903414	AMY1A	AK292341	0,004945	1,3646

25308	8107769	SLC12A2	AY280459	0,024875	1,36784
31167	8162940	ABCA1	NM_005502	0,019846	1,37285
15086	8005689	LOC101060794	ENST00000413270	0,019536	1,40336
22999	8085058	AC090043.1	OTTHUMT00000337388	0,027961	1,40882
19759	8052382	FANCL	BC037570	0,001728	1,45387
20541	8060736	PANK2	BC008667	0,024818	1,47896
17241	8027429	CTC-448F2.4	OTTHUMT00000459473	0,033898	1,54452
13387	7987464	RP11-521C20.1	OTTHUMT00000418283	0,027505	1,59838
12764	7981781	OR4M2	BC136985	0,044825	1,63658
6379	7918768	DENND2C	NM_001256404	0,024137	1,65674

Supplemtary Table IV

			CTR		RE		RVE								
**Pathway**	**Swiss-Prot accession number**	**Gene Symbol**	**Fc (post vs pre)**	**p-value**	**Fc (post vs pre)**	**p-value**	**Fc (post vs pre)**	**p-value**	**MASCOT protein score**	**Coverage (%)**	**Matched/ searched peaks**	**MS/MS**	**MS/MS score**	**m/z**	**z**
**Glycolysis**	Q5U077	LDHB	1,27	0,0169	-1,54	1,42E-03	1,03	0,257	156	33,5	11/18	**159**VIGSGCNLDSAR**170**	83	1248,617	1
	P11177	PDHB	1,55	5,69E-03	-1,19	2,83E-03	1,06	1,12E-01	104	31,8	12/21	**325**VTGADVPMPYAK**336**	31	1264,626	1
**Citrate cycle (TCA cycle)**	P48735	IDH2	1,9	6,65E-03	-1,16	3,18E-03	-1,1	5,75E-02	114	25,4	12/30	**341**TIEAEAAHGTVTR**353**	121	1355,724	1
	P48735	IDH2	1,25	0,0239	-1,25	2,83E-03	-1	0,326	131	35	14/38	**341**TIEAEAAHGTVTR**353**	118	1355,724	1
**Malate shuttle**	P17174	GOT1	1,17	0,12	-1,06	0,0432	-1,02	0,276	84	16,9	7/15	**294**ITWSNPPAQGAR**305**	55	1297,693	1
	P17174	GOT1	1,07	0,209	-1,13	0,0302	-1,04	1,48E-01	105	17,4	7/9	**294**ITWSNPPAQGAR**305**	72	1297,673	1
	P17174	GOT1	1,27	0,0319	-1,28	2,44E-03	-1,05	4,93E-02	172	34,1	13/22	**294**ITWSNPPAQGAR**305**	90	1297,681	1
	P40925	MDH1	1,32	0,0214	1,17	8,24E-03	-1,06	1,48E-01	104	23,1	7/9	**299**FVEGLPINDFSR**310**	70	1393,760	1
	P40925	MDH1	1,01	0,399	-1,13	0,0278	-1,12	3,41E-02	110	30,5	9/17	**299**FVEGLPINDFSR**310**	60	1393,725	1
	P40925	MDH1	1,97	3,46E-03	-1,2	6,96E-03	-1,04	7,42E-02	102	34,1	9/22	**299**FVEGLPINDFSR**310**	117	1393,727	1
	P00505	GOT2	1,63	2,83E-03	-1,06	0,06	1,4	1,47E-02	122	19,3	14/27	**69**DDNGKPYVLPSVR**81**	88	1459,740	1
**Oxidative phosphorylation**	P25705	ATP5A1	1,22	0,083	-1,28	7,55E-04	-1,01	0,297	93	18,1	10/18	**335**EAYPGDVFYLHSR**347**	103	1553,737	1
	P06576	ATP5B	-1,16	0,123	-1,34	3,65E-03	-1,15	3,17E-02	165	38,2	11/15	**162**AHGGYSVFAGVGER**175**	84	1406,675	1
	P06576	ATP5B	1,4	0,0152	-1,28	2,23E-03	-1,09	4,69E-02	238	49,4	21/30	**162**AHGGYSVFAGVGER**175**	109	1406,695	1
**Lipid Metabolism**	P49748	ACADVL	1,69	7,29E-03	-1,4	3,97E-03	-1,01	0,294	136	25,6	17/41	**588**SLSEGHPTAQHEK**600**	73	1420,689	1
**Translation**	P49411	TUFM	1,01	0,424	-1,24	6,97E-03	-1,06	9,06E-02	111	22,6	10/21	**91**KYEEIDNAPEER**102**	94	1492,703	1

Supplementary figure legends

Supplementary Table I: List of all differentially regulated genes in CTR group (end vs. pre). The differentially regulated genes meeting $p < 0.05$ and < -1.3 & >1.3 fold change criteria are included in the table. The list includes only annotated genes.

Supplementary Table II: List of all differentially regulated genes in RE group (end vs. pre). The differentially regulated genes meeting $p < 0.05$ and < -1.3 & >1.3 fold change criteria are included in the table. The list includes only annotated genes.

Supplementary Table III: List of all differentially regulated genes in RVE group (end vs. pre). The differentially regulated genes meeting $p < 0.05$ and < -1.3 & >1.3 fold change criteria are included in the table. The list includes only annotated genes.

Supplementary Table IV: Protein identifications by mass spectrometry. Spots were identified by MALDI/MS. To confirm identification, a MS/MS spectrum per protein was collected by Ultraflex III MALDI-ToF/ToF mass spectrometer.

Supplementary Figure 1

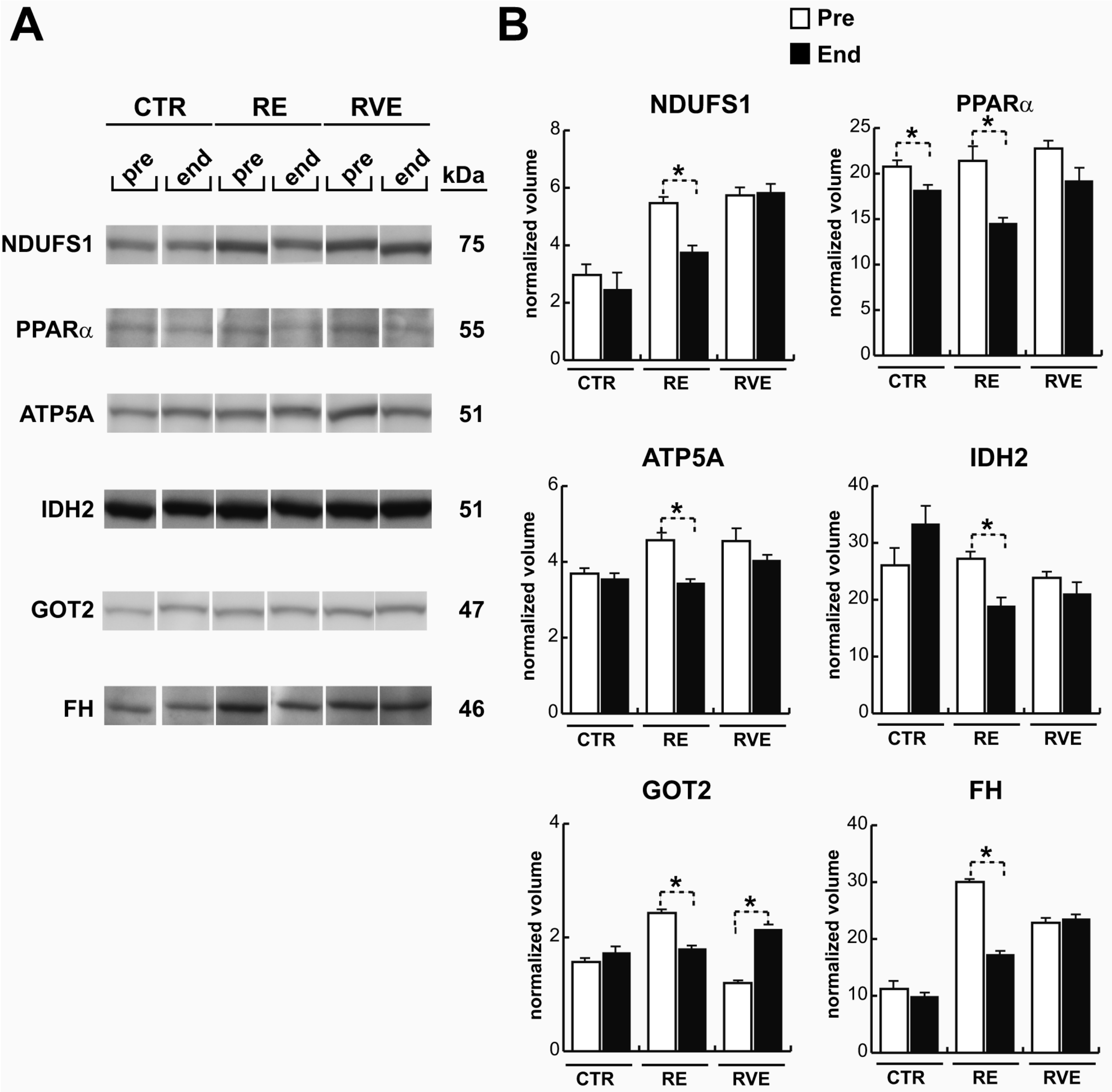

Fig.1S Array and 2D-DIGE orthogonal validation by immunoblotting of selected key muscle proteins with a differential expression pattern in CTR pre- and end-, RE pre- and end-, RVE pre- and end- in SOL skeletal muscle of the investigated subjects. Protein extract (50 μg), from samples analyzed were separated in triplicate in SDS-PAGE gel and transferred at 300 mA for 180 min to PVDF membranes. The membranes were incubated with rabbit or goat polyclonal primary antibodies (Santa Cruz Biotechnology except when differently stated) as follows: anti-NADH dehydrogenase (ubiquinone) Fe-S protein 1 (NDUFS1), 1:500; anti-peroxisome proliferator-activated receptor alpha (PPARα), 1:500; anti-ATP synthase 5A (ATP5A1), 1:500; anti-isocitrate dehydrogenase 2 (IDH2), 1:500; anti-glutamic-oxaloacetic transaminase 2, (GOT2, Bioss Antibodies), 1:1000; anti-fumarate hydratase (FH), 1:500. After washing, membranes were incubated with anti-rabbit (GE Healthcare) or anti-goat secondary antibodies conjugated with horseradish peroxidase. The signals were visualized by chemiluminescence using the ECL Plus detection kit. Image analysis (Image Quant TL; Molecular Dynamics, Sunnyvale, CA, USA) was performed followed by statistical analysis (paired Student's t test, $P<0.05$). The data were normalized against the total amount of proteins stained by Sypro Ruby (Molecular Probes).

(A) Shows the membrane close-up.

(B) Shows quantitative results.
